# Simulation of realistic patella fractures: an investigation into the mechanism and potential benefit for surgical training

**DOI:** 10.1186/s43019-025-00281-6

**Published:** 2025-07-14

**Authors:** Sebastian Wegmann, Jannik Leyendecker, Tim Leschinger, Maximilian Weber, Lars-Peter Mueller, Andreas Harbrecht

**Affiliations:** Faculty of Medicine and University Hospital, Center for Orthopedic and Trauma Surgery, University of Cologne, Kerpener Str. 62, 50937 Cologne, Germany

## Abstract

**Introduction:**

Patella fractures account for about 1% of all bone fractures, predominantly affecting males at a 2:1 ratio and exhibiting distinctive age-related patterns. In younger individuals, these injuries typically result from high-velocity impacts, while in the elderly, they usually arise from lower-energy impacts. Consequently, the types of fractures differ; horizontal fractures are more common in younger individuals, whereas comminuted fractures are more prevalent in older adults. Owing to the knee’s biomechanics, surgical intervention is often necessary. Preserving the articular surface is crucial to prevent retropatellar osteoarthritis, making thorough planning of surgical treatment essential. How can the osteosynthesis of this fracture entity be simulated as realistically as possible?

**Materials and methods:**

This study focused on the feasibility of inducing realistic patella fractures with an intact soft tissue envelope on human cadaveric specimens for surgical training purposes. A total of seven fresh-frozen human cadaveric knee joints were used, and fractures were created using a custom-designed drop-test bench. The induced fractures were then classified according to the Arbeitsgemeinschaft für Osteosynthesefragen (AO) and Speck and Regazzoni classifications using radiographic and computed tomography (CT) evaluations. In addition, intra-rater and inter-rater reliability were further examined.

**Results:**

All specimens were successfully fractured. The results demonstrated high intra-rater and inter-rater reliability in both fracture classification systems, indicating that the method can reliably replicate realistic fractures for training purposes.

**Conclusions:**

The study highlights the significance of using specimens with realistically induced fracture patterns in surgical education. Given that patella fractures are relatively rare and limit direct clinical exposure, realistic fracture models are invaluable for understanding these conditions. These models enhance surgical training, enabling both novice and experienced surgeons to refine their skills and effectively adapt to new surgical techniques.

## Introduction

With a prevalence of approximately 1% of all fractures, patella fractures are considered a rare pathology [1, 2]. Male patients are more commonly affected by injuries of the patella when compared to females, with a ratio of approximately 2:1 [3]. Age distribution cluster patients into two groups with distinct pathophysiological differences. Younger patients predominantly present with horizontal fractures, while comminuted fractures are more commonly found in elderly patients around 60 years of age. In younger patients, these fractures usually result from high-velocity traumas, whereas in the elderly, they are due to low-energy traumas. The differences in fracture morphology arise from the higher fragility of ageing bone [4]. Despite the relatively small layer of soft tissue covering the patella, open fractures occur in only about 1 in 14 fractures [3]. The most common cause of patella fractures is road, work or domestic accidents through direct or indirect trauma [5]. In cases of indirect trauma, the force exerted by the extensors exceeds the tensile strength of the patella, frequently resulting in transverse fractures. Conversely, direct impact to the patella with the knee in a flexed position most commonly leads to the patella’s inability to withstand compressive forces, resulting in a comminuted fracture [6, 7]. 

Most patella fractures require surgical treatment to re-establish the knee’s biomechanics. However, perioperative complications, especially wound disruptions and infections, frequently occur. Furthermore, a relevant number of patients suffer from persistent functional impairment and sustained pain [8, 9]. Geriatric patients are especially susceptible to prolonged hospitalisation, readmission and reoperation [10]. This likely originates from the relatively thin soft tissue covering the patella and its proximity to the femur. As a result, significant articular cartilage damage can occur even with seemingly minimal fractures [11]. Moreover, the overall low prevalence of patella fractures results in low case numbers for the respective surgeon and, thus, inexperience [1, 12, 13].

Hence, practising operative procedures for patella fractures in a controlled environment with life-like pre-fractured specimens may be essential. These simulated surgeries offer surgeons invaluable experience and exposure, ultimately optimising outcomes for patients with patella fractures. Offering courses with pre-fractured specimens can significantly enhance surgical training [14].

Despite being time-consuming, cost-intensive and complex, practical surgical training is essential for the education of aspiring and experienced surgeons [15].

Today, many educational concepts for surgeons are available, including virtual reality simulations and various simulators. The predominant idea in orthopaedic and trauma surgery involves using artificial bone samples that mimic human anatomy. However, these samples lack soft tissue coverage in fractures induced by osteotomies. In essence, human cadaveric specimens offer a more realistic training experience, providing a life-like model for both surgical approach and fracture reduction and fixation. This study explores the feasibility of inducing life-like patella fractures in human cadaveric specimens, thus addressing the need for realistic simulations in surgical training.

## Material and methods

### Specimen characteristics and preparation

For the present study, seven fresh-frozen human cadaveric knee joints were available. The fractures were induced using a custom-made drop-test bench.

The specimens were obtained from body donors, and written consent was obtained before death. All procedures performed in our study involving human cadavers were conducted by the ethical standards of the local ethics committee and by the 1964 Declaration of Helsinki and its later amendments or comparable ethical standards. Institutional ethics committee approval was obtained prior to this study (23–1184).

The donors had an average age of 68 years (range 62–78 years) at the time of death, comprising three males and four females. Fractures were induced in four proper and three left knee joints, with one specimen having fractures in both patellae. Before testing, X-ray images were examined for degenerative changes, sequelae from previous fractures, or the presence of implants that could affect fracture simulation, which excluded such specimens. Following a standardised protocol for all specimens, the femur was detached 10 cm above the patella, while the tibia and fibula were detached 10 cm below. The remaining soft tissue was left intact without interference. Closure of the soft tissue at the amputation site was performed using a 2–0 polydioxanone suture (PDS) wire.

### Test-bench and fracture simulation

**Fig. 1:**
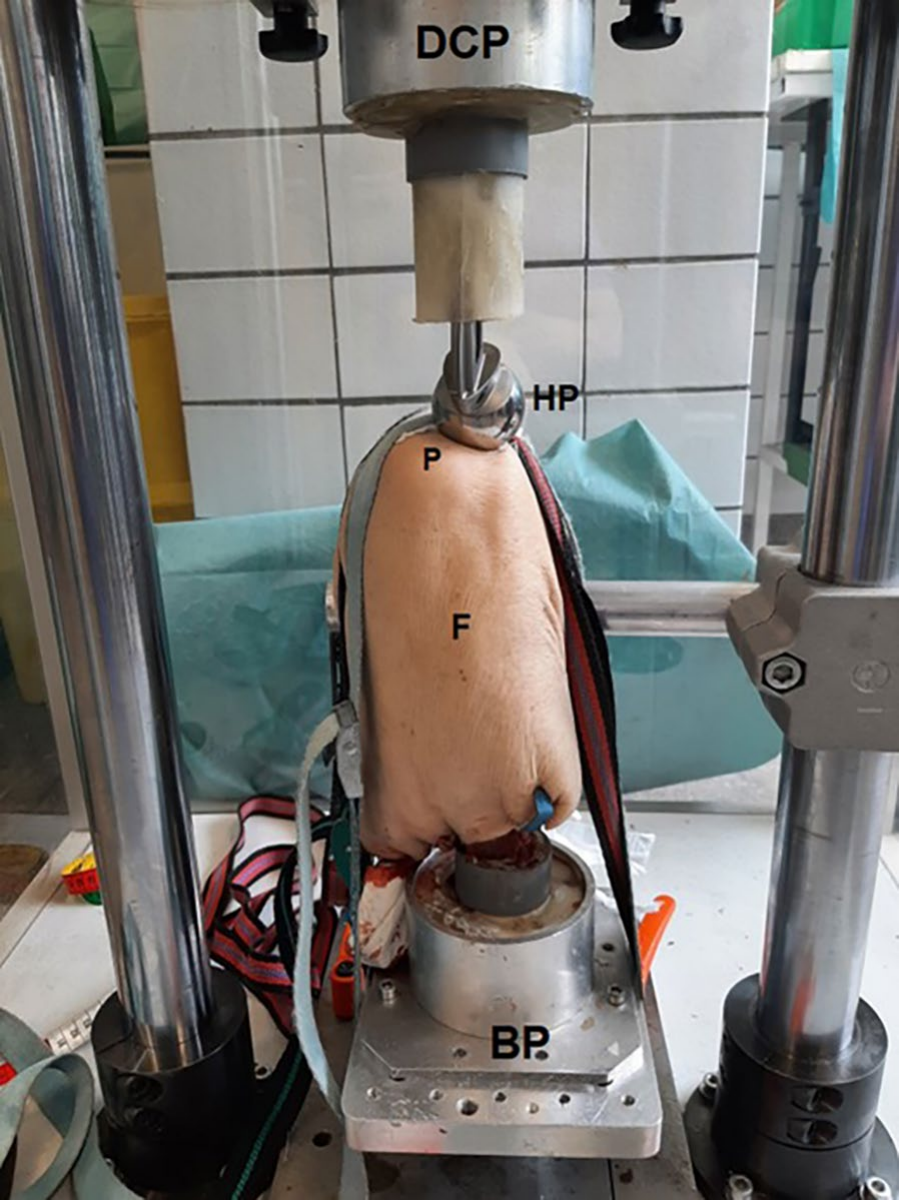
Typical drop-test bench for patella fracture simulation: impact was performed via a hip hemiprothesis (HP), which was mounted to the distal contact plate (DCP) with the femur (F) and tibia (not visible) potted into a custom-made steel cylinder with polymethyl methacrylate (PMMA) fixed to the base plate (BP). The specimen and the patella (P) were additionally fixed with tension bands

After thawing at room temperature, the specimens were placed supine on the test bench, with their patellae facing upward. The femur and tibia were anchored to the base plate using tension bands, and similar stabilisation was applied to the patella during the impact process (Fig. 1). A custom-made drop-test bench was utilised to apply the necessary force for fracture induction, a method in line with previous studies conducted by the research team [16, 17].

**Fig. 2:**
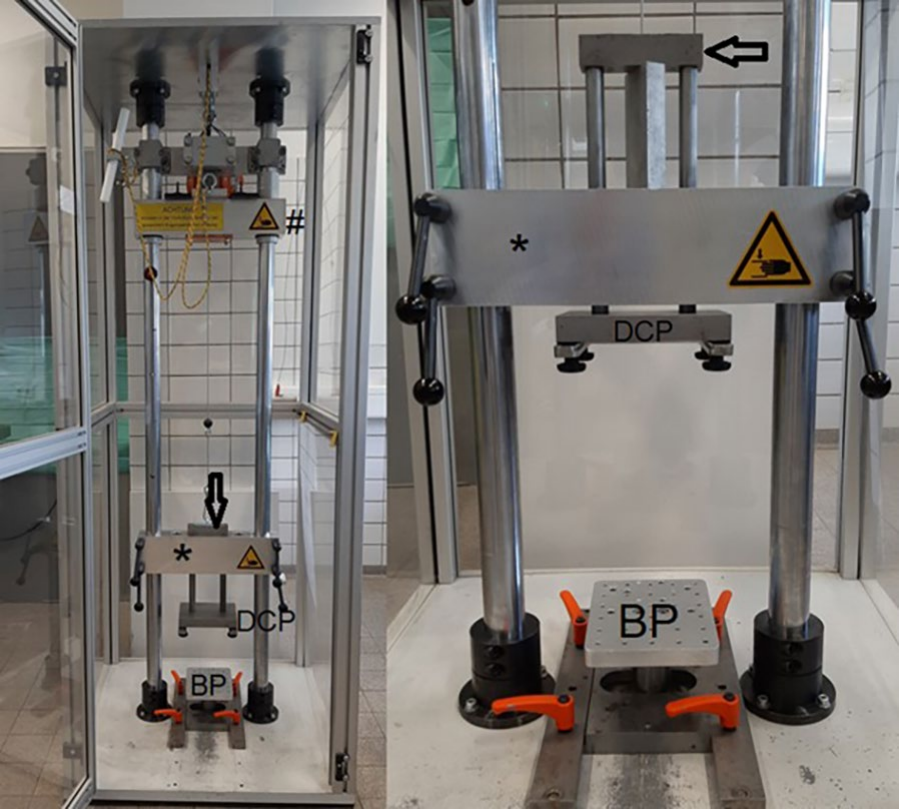
Custom-made drop-test bench – unloaded. The drop weight (#) impacts the crossbeam (*), which in turn pushes the upper contact plate (arrow) downwards and transfers kinetic energy to the specimen, which is fixed to the baseplate (BP). In our case, a hip hemiprosthesis was mounted onto the distal contact plate (DCP)

**Fig. 3:**
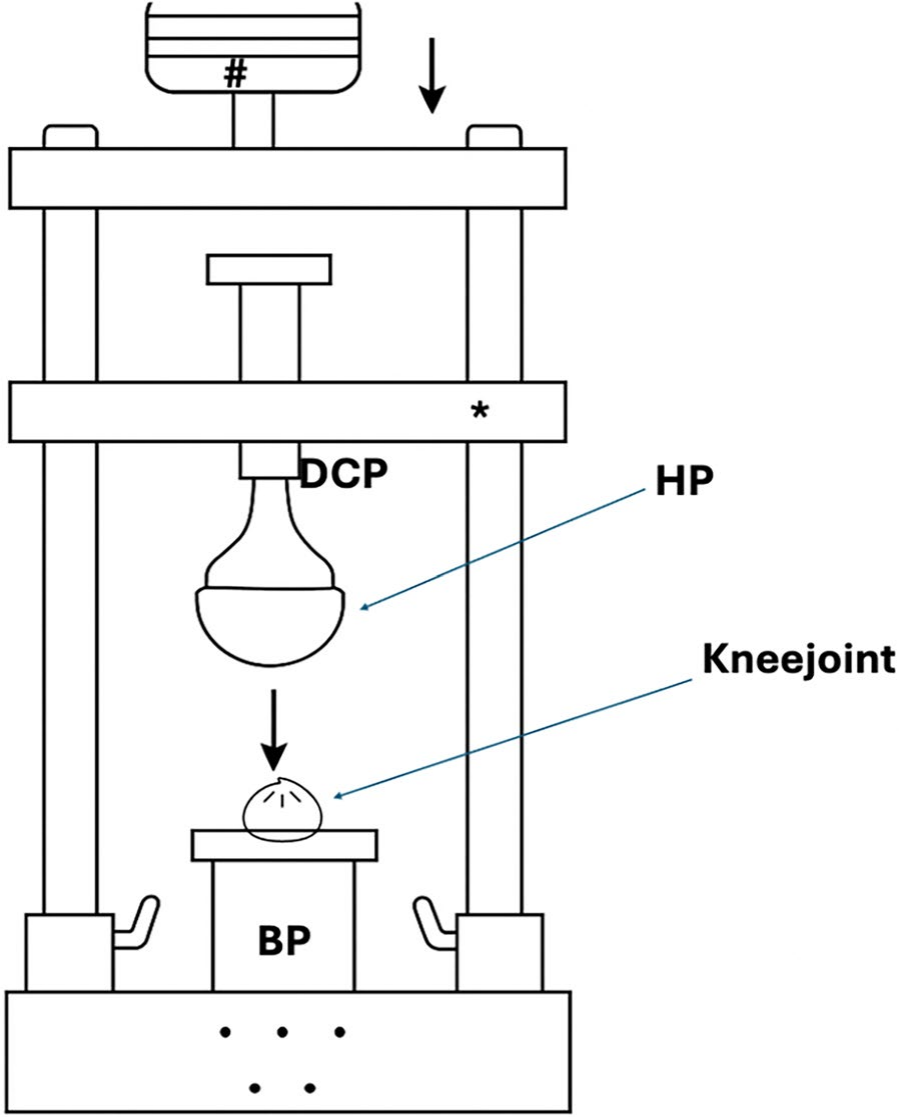
Schematic drawing of the custom-made drop-test bench. The drop weight (#) will impact the crossbeam (*), which will push the upper contact plate distally and transfer kinetic energy to the distal contact plate where the hemiprosthesis (HP) is mounted. The kinetic energy will be transferred into a downward movement of the prosthesis, which will then impact the knee joint mounted on the baseplate (BP)

The custom-made drop-test bench includes a steel framework mounted on a 15-mm thick steel baseplate, measuring 1.5 units on each side, ensuring stable positioning of the test bench and uniform force distribution. The frame is encased in a height-adjustable crossbeam and an impact beam. The crossbeam features two slide holes for inserting two stems into an impact stamp equipped with two contact plates – one above and one below the crossbeam. The potting cylinder’s base plate, bearing the impact stamp, can be affixed to the proximal contact plate (Fig. 1). By levelling the adjustable crossbeam, the amount of overhang of the impacting stamp above the crossbeam can be set. When the impacting beam is dropped onto the upper contact plate of the impacting stamp, it is pushed downwards, compressing the specimen by a magnitude correlating to its protrusion above the crossbeam. The compression of the specimen then induces the required fracture (Fig. 2). Figure 3 shows a schematic drawing of the bench for easier understanding. The apparatus can achieve an impact velocity of up to 210 Joules (*J*) of kinetic energy (*E* = 1/2 *mv*^2^; *E* = energy, *m* = mass, *v* = velocity), calculated at an impact speed of 4 m/s [18].

To produce patella fractures, a hip hemiprothesis (Austin Moore prosthesis, BIONTECH, Garbsen, Germany, head size 40 mm) was potted (polymethyl methacrylate—PMMA) into a custom-made steel cylinder with the prosthesis facing downwards to the baseplate of the test bench after it was attached to the distal contact plate. Then, the patella was positioned on the baseplate to allow the distal contact plate with the prosthesis to fit onto the patella orthogonally (Fig. 1).

After the fracture induction, radiographs were taken, and further evaluation was done through computed tomography (CT) imaging.

### Classification

Two board-certified trauma surgeons (S.W., J.L.) classified all fractures independently using the frequently used Arbeitsgemeinschaft für Osteosynthesefragen (AO) and Speck and Regazzoni classifications (Tables 1 & 2) [5, 13, 19].

**Table 1:** Overview of the AO classification for patella fractures

34	Type A – extraarticular	Type B – partially articular/sagittal fracture	Type C – complete articular/frontal or coronal fracture
1	Avulsion fracture	Longitudinal lateral, simple Longitudinal lateral, comminuted	Oblique (middle third) Oblique (proximal third) Oblique (distal third)
2	Isolated patellar body	2.1. Longitudinal medial, simple 2.2. Longitudinal medial, comminuted	Oblique with additional fragment (wedge)
3			Complex, comminuted

The prefix 34 indicates that the classification refers to the patella. Type A fractures are extra-articular and are either avulsion fractures (A1) or isolated body fractures (A2). Type B fractures are partially articular fractures that run sagittally. B1 fractures are lateral longitudinal and B2 fractures are medial longitudinal. Type C fractures are completely articular fractures, C1 fractures are oblique, C2 fractures are oblique with an additional fragment and type C fractures are complex and comminuted

**Table 2:** Overview of the Speck and Regazzoni classification for patella fractures

	Type A – Longitudinal fracture	Type B – Oblique fracture	Type C – Comminuted
1	Non-displaced	Pole avulsion without joint involvement (upper pole > 5 mm, lower pole > 15 mm)	Without dislocation
2	Displaced	Simple	With dislocation (> 2 mm)
3	With additional fragment	With additional fragment	Dislocation with burst pattern

Our institution’s picture archiving and communication system (IMPAX) was used to calculate Hounsfield units (HU) as a reference for bone mineral density in specimens that underwent computed tomography (CT) scanning. Feld [20–25]. According to Wagner et al., measurements were performed on three consecutive coronal CT slices at the level of the patella [26]. The region of interest was measured using a circle to best fit the femur, excluding the cortical surface. The mean of three measurements was then calculated for each specimen [27].

### Statistical analysis

To assess the reliability of the fractures, Pearson correlation coefficients (*r*) were calculated for both intra-rater and inter-rater reliability using Excel (Microsoft Corporation, 2018). For intra-rater reliability, the classification dates were spaced 8 weeks apart. Descriptive statistics were utilised to summarise the means and standard deviations. A significance level of *p* < 0.05 was set.

## Results

All the specimens could be fractured successfully by our custom-made drop-test bench. Table 3 summarises the results of the induced fractures.

**Table 3:** Individual characteristics of each specimen

Specimen	AO classification	Speck and Regazzoni classification	CT scan (HU)	Gender	Age	side
C170928	34 C2	B3	272	Male	78	Right
C171246	34 C3	C2	356	Male	66	Right
C190931	34 C2	B3	282	Female	76	Left
I190607	34 B1.2	A3	291	Female	62	Left
I190764	34 C2	B3	261	Female	63	Left
I190764	34 A1	B1	256	Female	63	Right
K-12–16	34 C3	C2	241	Male	70	Right

### Hounsfield units

The mean HU was 295 (range 146–356).

### Intra-rater correlation

The intra-rater correlation coefficient for the AO classification system and the Speck and Regazzoni classification was *r* = 1 with a *p* < 0.001, indicating a perfect agreement.

### Inter-rater correlation

The inter-rater correlation coefficient was perfect for both classification systems. The AO classification documented a coefficient of *r* = 0.99 (*p* < 0.001) and the Speck and Regazzoni classification for *r* = 0.85 (*p* = 0.02).

## Discussion

The study showcases the feasibility of inducing life-like patella fractures in human cadaveric specimens. Despite the technically demanding and costly nature of the process, creating realistic fractures holds promise for providing invaluable educational benefits to trauma surgeons.

Patella fractures represent only a fraction of all human fractures, thus limiting surgical exposure and experience in their treatment. Therefore, both residents and experienced surgeons stand to gain from realistic fracture models to enhance their skill sets. All induced fractures exhibited high clinical relevance. The testing setup facilitated the reproducibility of fractures, primarily through direct trauma, with a notable prevalence of comminuted fractures. These aspects are particularly relevant for experienced surgeons seeing more complex fracture types [6, 7].

Since new surgical techniques and implants are introduced frequently, reproducible fracture models would facilitate a realistic setting, enabling surgeons to familiarise themselves with new treatment options. Thus far, this setting has been validated for other fractures. A broader spectrum of fractures and patterns, now including patella fractures, enables the installation of a comprehensive training system for excellent surgical education. Furthermore, we achieved a good inter-rater and intra-rater correlation, which supports the recommended utilisation of the AO classification system and the Speck and Regazzoni classification [5].

The authors acknowledge that this study has limitations. Most importantly, the simulation setup would benefit from further evaluation. In the current study, transverse fractures commonly seen in indirect trauma could not be induced using the presented method. The drop-test bench only simulates a direct impact on the patella owing to the necessity of stable positioning and fixation of the knee joint under an accurate impactor, such as a hip hemiprosthesis. Surrounding ligaments, especially the quadriceps and patella tendons, could not be simulated adequately in a cadaveric setting. As transverse fractures account for a large proportion of patella fractures, unfortunately, these cannot be replicated by the current experimental setup. However, from a technical perspective, these fractures are less demanding intraoperatively, whereas multi-fragmentary fractures present a greater surgical challenge.

Furthermore, alterations in axial weight load and height might extend the range of fracture complexity. Lastly, the included specimens in this study originated from elderly patients. Thus, the group of young patients suffering from high-velocity trauma is under-represented. As the survey by Narayanan et al. demonstrates, elderly patients with lower Hounsfield units, and therefore lower bone mineral density, have a higher risk of fractures. This could also be shown for the proximal humerus; however, Hounsfield units cannot be used as a predictive factor for comminuted fractures [28].

For the future, the objective should be to evaluate the feasibility and impact of the model using pre- and post-training assessments, procedural error counts or time-to-completion metrics.

## Conclusions

Reproducible and reliable induction of patella fractures in human cadaveric specimens is feasible. The presented findings have the potential to become a cornerstone in the development of individualised educational training for both aspiring and experienced surgeons, as the results of this study are valuable teaching models with realistic patella fracture patterns.

## Data Availability

Data sharing does not apply to this article as no datasets were generated or analysed during the current study.
